# Eugenol Mitigates Mercuric Chloride–Induced Renal Injury by Attenuating Oxidative Stress, Ferroptosis, ER Stress, Apoptosis, and Autophagy

**DOI:** 10.1007/s12011-026-05115-4

**Published:** 2026-04-29

**Authors:** Samet Teki̇n, Özlem Erol POLAT, Merve Bolat, İsmail Bolat, Betül Orhan, Ömercan Alat, Burak Batuhan Laçi̇n, Furkan Aykurt

**Affiliations:** 1 Department of Physiology, Faculty of Veterinary Medicine, Atatürk University, Erzurum, Türkiye; 2 Department of Pathology, Faculty of Veterinary Medicine, Atatürk University, Erzurum, Türkiye; 3 Department of Biochemistry, Faculty of Veterinary Medicine, Atatürk University, Erzurum, Türkiye

**Keywords:** Mercuric chloride, Eugenol, Ferroptosis, Nephrotoxicity, Endoplasmic reticulum stress, Sprague–Dawley rat

## Abstract

This study aimed to comprehensively evaluate the effects of eugenol (EUG) on oxidative stress, inflammation, ferroptosis, endoplasmic reticulum (ER) stress, autophagy, and apoptosis in an experimental nephrotoxicity model induced by mercury chloride (HgCl₂). Thirty male Sprague–Dawley rats were divided into five groups: control, EUG (100 mg/kg), HgCl₂ (1.23 mg/kg), HgCl₂+EUG50, and HgCl₂+EUG100. HgCl₂ was administered intraperitoneally, and EUG was administered orally for 7 days. Oxidative stress and inflammation parameters in kidney tissues were evaluated by ELISA; histopathological changes were evaluated by H&E and Masson’s Trichrome staining; ferroptosis markers (GPx4, ACSL4, FTH1) were evaluated immunohistochemically; and DNA oxidative damage (8-OHdG) was evaluated by immunofluorescence. Apoptosis-related proteins were analyzed by Western blot, while autophagy and ER stress-related genes were analyzed by RT-qPCR. HgCl₂ administration increased MDA levels in kidney tissue while significantly decreasing GSH and SOD levels; proinflammatory cytokines (TNF-α, IL-1β, IL-6) increased while IL-10 levels were suppressed. Histopathologically, marked tubular degeneration, necrosis, and fibrosis developed. In the HgCl₂ group, GPx4 and FTH1 expression decreased, while ACSL4 and 8-OHdG levels increased. Bax and caspase-3 protein levels were elevated, and Bcl-2 was suppressed. Additionally, autophagy (Beclin-1, LC3A/B) and ER stress (eIF2α, ATF4, CHOP, IRE1) gene expressions were significantly elevated. EUG administration, particularly at a dose of 100 mg/kg, significantly improved these alterations; it reduced oxidative stress and inflammation, attenuated ferroptosis and ER stress–related alterations, and regulated apoptosis and autophagy responses. The findings reveal that eugenol exhibits a multi-pathway protective profile against HgCl₂-induced kidney damage, acting as an antioxidant, anti-inflammatory, and modulator of cellular stress responses. Eugenol represents a promising therapeutic candidate against heavy metal-induced nephrotoxicity.

**Supplementary Information:**

The online version contains supplementary material available at 10.1007/s12011-026-05115-4.

## Introduction

Mercury (Hg) is a toxic heavy metal that is widely found in the environment and enters it as a result of human-induced processes such as industrial activities, mining, dental amalgams, pesticide residues, and the burning of fossil fuels. In particular, mercury chloride (HgCl₂), the inorganic form of mercury, causes multiple organ damage in living organisms due to its high bioreactivity. HgCl₂’s high binding capacity to plasma proteins causes it to accumulate in many organs, primarily the liver, kidneys, and brain. The kidneys play a fundamental role in the excretion of mercury from the body and are therefore among the tissues most affected by HgCl₂ exposure [1–3].

HgCl₂ causes significant nephrotoxicity, characterized by mitochondrial dysfunction, ion imbalance, and increased ROS production, particularly in proximal tubular cells. Increased H₂O₂ production originating from the mitochondria also contributes to this condition [4, 5]. Excessive ROS accumulation triggers lipid peroxidation and leads to suppression of antioxidant defense systems (GSH and SOD) [6, 7]. In this oxidative stress environment, GPx4 expression and activity are suppressed by Hg²⁺; this inhibits the breakdown of lipid hydroperoxides and directs the cell toward ferroptotic death [8]. HgCl₂ can also cause intracellular iron accumulation by affecting iron homeostasis; specifically, decreased ferritin levels increase the free Fe²⁺ pool, thereby increasing hydroxyl radical production via the Fenton reaction. A model has demonstrated that the decrease in ferritin (especially FTH1) expression over time with mercuric chloride administration may lead to iron release via ferritinophagy [9]. These radicals cause oxidative damage to DNA and increase the formation of 8-OHdG [6]. In addition, increased ROS production increases the production of proinflammatory cytokines such as TNF-α, IL-1β, and IL-6. This inflammation also supports the apoptosis signal [10, 11]. An increase in the Bax/Bcl-2 ratio triggers cellular apoptosis by disrupting mitochondrial membrane permeability through caspase-3 activation [12].

HgCl₂ also triggers endoplasmic reticulum (ER) stress. In this context, exposure to HgCl₂ has been found to activate PERK, ATF-6, and IRE1 signaling pathways in kidney tissues [13]. The PERK pathway suppresses overall protein synthesis by increasing eIF2α phosphorylation and induces the ATF4–CHOP complex during prolonged stress. CHOP promotes apoptosis via the mitochondrial pathway by suppressing Bcl-2 and activating Bax [14, 15]. The IRE1 arm increases protein folding capacity through XBP1 splicing, while excessive activation may support pro-apoptotic signals. ATF6 translocates to the nucleus through proteasomal activation and increases the expression of ER chaperone genes [15]. Therefore, mercury-induced kidney toxicity results from the complex interactions between oxidative stress, inflammation, ER stress, and apoptosis [16].

In recent years, the importance of plant-derived natural compounds in preventing tissue damage caused by toxic agents has been increasing [17]. Phenolic compounds maintain cellular redox balance by neutralizing free radicals thanks to their hydroxyl groups, can chelate metal ions, and can suppress inflammatory signaling [18]. One of these compounds, eugenol (EUG; 4-allyl-2-methoxyphenol), is a natural monoterpene phenol found in many plants, primarily cloves (Syzygium aromaticum) [19]. The antioxidant and anti-inflammatory effects of EUG demonstrate its protective potential against cellular damage caused by oxidative stress and inflammation [17]. EUG directly neutralizes free radicals via the phenolic hydroxyl group while increasing the expression of antioxidant enzymes by activating the Nrf2/Keap1/HO-1 pathway [20]. EUG can reduce the production of proinflammatory cytokines such as TNF-α, IL-1β, and IL-6 by suppressing the NF-κB and MAPK inflammatory signaling pathways [17]. This multifaceted mechanism of action suggests that EUG may play a protective role in cellular damage models caused by oxidative stress, inflammation, and apoptosis [21].

The protective effects of EUG in different organ toxicities have been previously identified in various experimental models [22]. EUG has been reported to reduce oxidative damage by supporting the antioxidant defense system in gentamicin-induced nephrotoxicity [23]. Previous studies have shown that EUG reduces cardiomyocyte damage by preventing oxidative stress in doxorubicin-induced cardiac (cardiovascular) toxicity [24].

In this study, the effects of two different doses of EUG (50 and 100 mg/kg, oral) in an experimental kidney injury model induced by HgCl₂ were evaluated using biochemical, histopathological, immunohistochemical, immunofluorescence, Western blot, and Real-Time PCR analyses. Changes in molecular markers associated with oxidative stress, inflammation, ER stress, and apoptosis were compared to investigate the potential protective effect of EUG. The obtained data suggest that EUG can be considered as a potential protective agent against HgCl₂-induced kidney toxicity.

## Materials and Methods

### Chemicals

The mercury chloride (HgCl₂, CAS No: 7487-94-7, ≥ 99.5% purity) used in this study was obtained from ISOLAB (Germany), while eugenol (EUG, CAS No: 97-53-0, ≥ 99% purity) was obtained from Sigma-Aldrich (St. Louis, MO, USA).

### Laboratory Animals and Experimental Design

The study used a total of 30 adult male Sprague-Dawley rats weighing 220–250 g over a 12-week period. The animals were obtained from the Atatürk University Medical Experimental Research Center (ATADEM). The rats were housed in a well-ventilated environment at 25 ± 2 °C with a 12-hour light/dark cycle and had ad libitum access to standard pellet feed and water. Ethical approval for this study was obtained from the Atatürk University Local Animal Experiments Ethics Committee (Decision No: 2025/12/278). All experimental procedures were performed in accordance with the European Union’s Animal Experiments Directive 2010/63/EU and ARRIVE (Animal Research: Reporting of In Vivo Experiments) guidelines.

A one-week adaptation period was provided for the animals prior to experimental procedures. Rats were randomly assigned to five groups (*n* = 6) (Table 1). The distribution of animals among groups was performed using a computer-based random number generation method prior to the experiment.

**Table 1:** Experimental groups and application methods

Groups	Application
Control	1 mL distilled water (oral, 7 days)
EUG 100	100 mg/kg EUG (oral, 7 days) [25]
HgCl₂	1.23 mg/kg HgCl₂ (i.p., 7 days) [26]
HgCl₂ + EUG 50	1.23 mg/kg HgCl₂ (i.p.) + 50 mg/kg EUG (oral, 7 days) [25, 26]
HgCl₂ + EUG 100	1.23 mg/kg HgCl₂ (i.p.) + 100 mg/kg EUG (oral, 7 days) [25, 26]

Eugenol, due to its hydrophobic nature, is insoluble in distilled water. Therefore, prior to each application, it was vortexed in distilled water to prepare a homogeneous suspension and administered via oral gavage. The suspension was freshly prepared before each administration to ensure uniform dosing. Animals in the control group received the same volume of distilled water (vehicle) via oral gavage as the experimental groups. All applications were performed once daily. Twenty-four hours after the last application, all rats were sacrificed under 2–3% sevoflurane inhalation anesthesia (Sevorane^®^; AbbVie, UK). Kidney tissues were rapidly removed and used for biochemical, histopathological, immunohistochemical, immunofluorescent, Western blot, and real-time PCR analyses.

After removal, the kidneys were washed with physiological saline. Right or left kidney samples taken from each animal were randomly selected for analysis to eliminate possible side differences. Some of the samples were fixed in 10% buffered formaldehyde for histopathological and immunohistochemical studies, while others were frozen in liquid nitrogen and stored at −80 °C for biochemical, Western blot, and molecular analyses.

### Biochemical Analyses

Levels of oxidative stress, antioxidant defense, and inflammatory response parameters in kidney tissues were determined using the enzyme-linked immunosorbent assay (ELISA) method on tissue homogenates. YL-Biont (Shanghai, China) commercial rat ELISA kits were used for the measurements: Rat Malondialdehyde (MDA) ELISA Kit (Cat. No: YLA0029RA), Rat Superoxide Dismutase (SOD) ELISA Kit (Cat. No: YLA0115RA), Rat Glutathione (GSH) ELISA Kit (Cat. No: YLA1511RA), Rat Interleukin-1β (IL-1β) ELISA Kit (Cat. No: YLA0030RA), Rat Interleukin-6 (IL-6) ELISA Kit (Cat. No: YLA0031RA), Rat Interleukin-10 (IL-10) ELISA Kit (Cat. No: YLA0440RA), and Rat Tumor Necrosis Factor-α (TNF-α) ELISA Kit (Cat. No: YLA0118RA). All procedures were performed according to the manufacturer’s instructions, and absorbance values were read at 450 nm wavelength using a microplate reader (BioTek EPOCH2, USA).

### Histopathological Examinations

At the end of the study, the kidney tissues obtained were fixed in a 10% neutral buffered formaldehyde solution. Following the fixation process, routine tissue follow-up procedures were applied, and the samples were embedded in paraffin blocks. Section 5 μm thick were prepared from the paraffin-embedded tissues using a microtome (Leica RM2235, Germany). The sections obtained were stained using the Hematoxylin-Eosin (H&E) and Masson’s Trichrome (MT) methods.

The stained preparations were examined under a Leica Flexacam i5 light microscope. H&E staining was used to evaluate degenerative and necrotic changes in epithelial cells as well as the presence of interstitial nephritis. MT staining was used to determine the distribution and severity of tissue fibrosis.

All histopathological evaluations were performed using the blind pathology method, and the severity of findings was classified as follows: absent (–), mild (+), moderate (++), severe (+++). The area of fibrosis detected by MT staining was also evaluated as a percentage and graded as follows: Grade 1: 0–5%, Grade 2: 5–15%, Grade 3: 15–25%, Grade 4: 25–50%, Grade 5: >50% [27, 28].

### Immunohistochemical Analyses

Following the routine tissue tracking procedure for immunohistochemical analyses, tissue sections mounted on poly-L-lysine-coated slides were deparaffinized and dehydrated. Endogenous peroxidase activity in the sections was then blocked by incubating them with 3% H₂O₂ for 10 min. Antigen retrieval was performed by heating in Tris-EDTA buffer (pH 9.0), and the sections were allowed to cool at room temperature. To prevent nonspecific background staining, the sections were incubated with a protein block solution for 5 min. The sections were then incubated with primary antibodies: GPx4 (Cat. No: DF6701, RRID: AB_2838663, rabbit polyclonal, 1:100), ACSL4 (Cat. No: DF12141, RRID: AB_2844946, rabbit polyclonal, 1:100), and FTH1 (Cat. No: DF6278 RRID: AB_2838244, rabbit polyclonal, 1:100) (Affinity Biosciences, China) were applied to the sections and incubated according to the manufacturer’s instructions. After the secondary antibody applications, 3-Amino-9-Ethylcarbazole (AEC) was added as a chromogen, and the stained sections were evaluated under a light microscope (Leica Flexacam i5) [29].

### Immunofluorescence Analysis

Tissue sections mounted on poly-L-lysine-coated slides for immunofluorescence analysis were deparaffinized and dehydrated. Antigen retrieval was performed by heating in citrate buffer (pH 6.1), and sections were allowed to cool at room temperature. To prevent nonspecific background staining, sections were incubated with protein block solution for 5 min. Subsequently, sections were incubated with the primary antibody 8-OHdG (Cat. No: ab10802, RRID: AB_297491, rabbit polyclonal, 1:100; Abcam) according to the manufacturer’s instructions. Texas Red-labeled anti-rabbit IgG (Cat. No: ab6719, RRID: AB_955597, 1:1000; Abcam) was used as the secondary antibody, and the sections were incubated in the dark for 45 min. Subsequently, 4′,6-diamidino-2-phenylindole (DAPI) (Cat. No: ab104140) containing mounting medium was added to the sections, which were then left in the dark for 5 min and covered with a coverslip. The stained tissues were examined under a fluorescence microscope (Zeiss Axio, Germany). Negative control sections were prepared without adding the primary antibody to evaluate nonspecific staining. Masson’s trichrome (MT), immunohistochemical (IHC), and immunofluorescent (IF) staining positivity was evaluated semi-quantitatively using the ImageJ analysis program. The staining intensity in the regions of interest (ROI) selected in the images obtained with the same microscope and camera settings for all groups was calculated as the percentage of stained area (area %) and the mean values obtained were used in statistical analyses. To enable reliable relative comparisons between groups, all images were recorded under standard and fixed imaging settings [30].

### Real-Time PCR Analyses

Total RNA was isolated from kidney tissues using QIAzol lysis reagent (Qiagen, Cat. No: 79306) according to the manufacturer’s protocol. The amount and purity of the obtained RNA were assessed using a NanoDrop spectrophotometer (BioTek Epoch, USA) based on the 260/280 nm ratio. Subsequently, complementary DNA (cDNA) synthesis was performed using the RT2 First Strand Kit (Qiagen, Cat. No: 330404). Gene expression analyses were performed using the RT2 SYBR^®^ Green qPCR Mastermix (Qiagen, Cat. No: 330500) and the target gene primers listed in Table 2 on a LightCycler 480 (Roche, Germany) device. PCR conditions were as follows: 10 min pre-denaturation at 95 °C, followed by a total of 45 cycles of 15 s at 95 °C and 1 min at 60 °C. Amplification specificity was confirmed by melting curve analysis. Gene expression levels were normalized to the β-actin reference gene and calculated using the 2^−ΔΔCT method (Table 2).

**Table 2:** Primer sequences used for RT-qPCR

Gene	Accesion Number	Sequence (5′−3′)
*LC3A*	NM_199500.2	F: GACCATGTTAACATGAGCGA R: CCTGTTCATAGATGTCAGCG
*LC3B*	NM_022867.2	F: GAGCTTCGAACAAAGAGTGG R: CGCTCATATTCACGTGATCA
*Beclin-1*	NM_053739.2	F: TCTCGTCAAGGCGTCACTTC R: CCATTCTTTAGGCCCCGACG
*eIF2-α*	NM_019356.1	F: AGACCTGGATATGGTGCCTA R: CCTTCGTAACCATAGCAAGC
*ATF-4*	NM_024403.2	F: CTTCCTGAACAGCGAAGTGT R: ATAGCCAGCCATTCTGAGGA
*CHOP*	NM_001109986.1	F: GAAGCCTGGTATGAGGATCT R: GAACTCTGACTGGAATCTGG
*IRE1*	NM_001191926.1	F: GCAGTTCCAGTACATTGCCATTG R: CAGGTCTCTGTGAACAATGTTGA
*Β-actin*	NM_031144.3	F: CAGCCTTCCTTCCTGGGTATG R: AGCTCAGTAACAGTCCGCCT

### Western Blot Analysis

Kidney tissues were homogenized on ice using RIPA lysis buffer (Santa Cruz Biotechnology, Cat. No: sc-24948) and centrifuged at 16,000 × g for 20 min at 4 °C. Protein concentrations in the resulting supernatants were determined using the BCA Protein Assay Kit (Thermo Pierce, USA), and equal amounts of protein (40 µg) were mixed with Laemmli sample buffer and denatured by heating at 95 °C for 5 min before being separated on 10% SDS-PAGE gels. After electrophoresis, the proteins were transferred to PVDF membranes (Thermo Scientific, USA), and the membranes were blocked with TBS-T solution containing 3% BSA. They were then incubated overnight at 4 °C with primary antibodies against β-tubulin (sc-5274, RRID: AB_843040), Bax (sc-20067, RRID: AB_626720), Bcl-2 (sc-7382, RRID: AB_626736), and Caspase-3 (sc-56053, RRID: AB_781829) (Santa Cruz Biotechnology, USA). After washing, membranes were incubated with HRP-conjugated secondary antibody (Santa Cruz, sc-2005) for 1.5 h at room temperature, bands were developed using Trident Femto HRP substrate (GeneTex), and visualized using the GelDoc XR (Bio-Rad, USA) system. The intensities of the protein bands were analyzed using Image Lab 6.1 (Bio-Rad) software, and the target protein signals were normalized to the β-tubulin value. The control group’s average was set to 1.0, and the results were calculated as fold change.

### Statistical Analysis

All quantitative data obtained from the study are expressed as mean ± standard deviation (SD). The normality of the data distribution was assessed using the Shapiro–Wilk test. For biochemical parameters (MDA, SOD, GSH, and cytokine levels) and gene expression data (Real-Time PCR) and protein expression analyses (Western blot, immunohistochemical and immunofluorescent ImageJ measurements) were performed using one-way analysis of variance (ANOVA), and Tukey’s multiple comparison test was applied in cases where significant differences were detected.

The Kruskal–Wallis test was used to analyze histopathological score data (H&E and Masson’s trichrome evaluations) that did not show a normal distribution, and the Dunn post hoc test was used for pairwise comparisons between groups.

All statistical analyses and graphical representations were performed using GraphPad Prism 10.6.1 (GraphPad Software, San Diego, CA, USA) software, and *p* < 0.05 was considered statistically significant. Statistical significance levels are indicated in the figures as follows: *p* < 0.05 (*), *p* < 0.01 (**), *p* < 0.001 (***); non-significant differences are indicated as “ns”.

## Results

### MDA and GSH Concentrations and SOD Protein Levels in Kidney Tissue

**Fig. 1:**
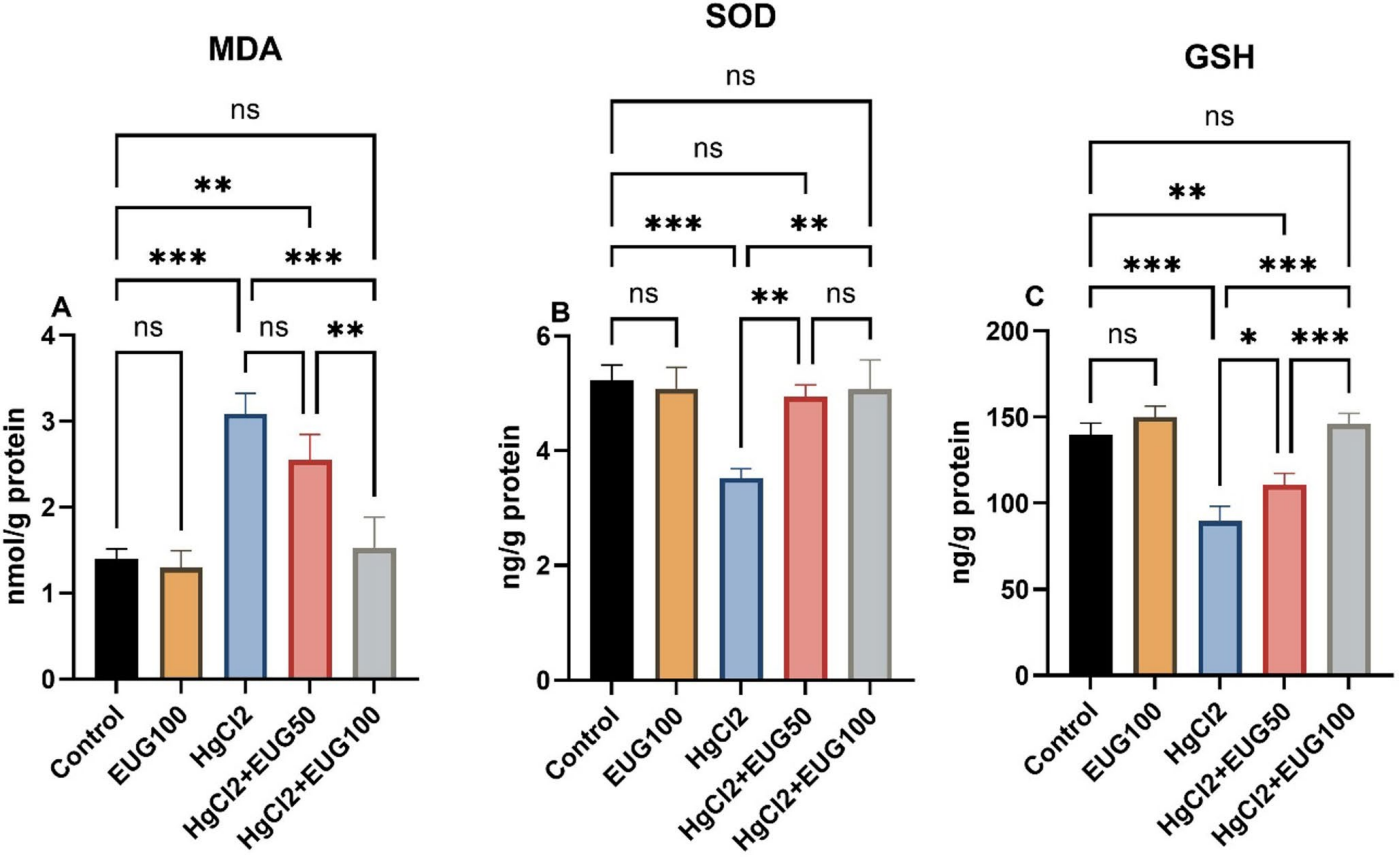
MDA and GSH concentrations (**A**, **C**) and SOD protein levels (**B**) in kidney tissue across the experimental groups. Data are presented as mean ± SD (*n* = 6). Statistical analysis was performed using one-way ANOVA followed by Tukey’s post hoc test. ***p < 0.001, **p < 0.01, *p < 0.05; ns, not significant

To evaluate the effects of HgCl₂ exposure on oxidative balance in kidney tissue and the potential protective role of EUG administration, biochemical parameters related to lipid peroxidation and the antioxidant defense system were analyzed. In this context, MDA and GSH concentrations and SOD protein levels in kidney tissue were examined, and the findings were compared between groups (Fig. 1).

The kidney MDA level in the HgCl₂ group was significantly higher than that of the control group at a level of *p* < 0.001. In the groups treated with EUG together with HgCl₂, the MDA level decreased significantly compared to the HgCl₂ group, and this decrease was particularly pronounced in the HgCl₂+EUG100 group at a level of *p* < 0.01 (Fig. 1).

The SOD protein level and GSH concentration in the HgCl₂ group showed a difference compared to the control group at the ***p < 0.001 level. When the HgCl₂ group was compared with the HgCl₂+EUG50 group, a difference was detected at the *p* < 0.01 level for SOD and at the *p* < 0.05 level for GSH. When comparing the HgCl₂ group with the HgCl₂+EUG100 group, a difference was found at the *p* < 0.01 level for SOD and at the *p* < 0.001 level for GSH, and it was determined that these parameters were higher in the groups treated with EUG (Fig. 1).

### Proinflammatory and Anti-Inflammatory Cytokine Levels in Kidney Tissue

**Fig. 2:**
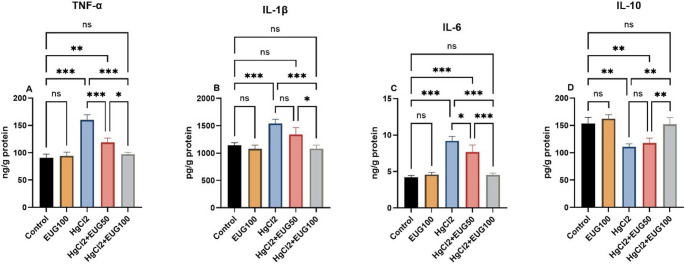
Levels of TNF-α (**A**), IL-1β (**B**), IL-6 (**C**), and IL-10 (**D**) in kidney tissue across the experimental groups. Data are presented as mean ± SD (*n* = 6). Statistical analysis was performed using one-way ANOVA followed by Tukey’s post hoc test. ***p < 0.001, **p < 0.01, *p < 0.05; ns, not significant

To evaluate the effects of HgCl₂ exposure on the inflammatory response in kidney tissue and the regulatory role of EUG administration on this response, proinflammatory and anti-inflammatory cytokine levels were analyzed (Fig. 2).

TNF-α, IL-1β, and IL-6 levels obtained from the groups treated with HgCl₂ were statistically different from those in the control group (*p* < 0.001). Values obtained from the HgCl₂+EUG50 group showed differences in TNF-α *p* < 0.001 and IL-6 *p* < 0.05 levels when compared to HgCl₂. When the values in the HgCl₂ group were compared with those in the HgCl₂+EUG100 group, statistically significant differences were found in TNF-α, IL-1β, and IL-6 levels at *p* < 0.001 (Fig. 2).

The IL-10 level in kidney tissue was found to be lower in the HgCl₂ group compared to the control at *p* < 0.01. When the values obtained from the HgCl₂ group were compared with HgCl₂+EUG100, a difference at *p* < 0.01 was determined between them (Fig. 2).

### Hematoxylin-Eosin and Masson’s Trichrome Staining Findings in Kidney Tissue Following HgCl₂ Application

Histopathological examinations revealed no significant pathological lesions in the kidney tissue of the control and EUG100 groups. In the HgCl₂ group, however, severe degenerative and necrotic changes were observed in the tubular epithelial cells. Although a decrease in these histopathological findings was observed in the HgCl₂+EUG50 group, no statistically significant difference was determined when compared to the HgCl₂ group. In the HgCl₂+EUG100 group, degenerative and necrotic changes were found to be statistically significantly reduced compared to the HgCl₂ group.

**Fig. 3:**
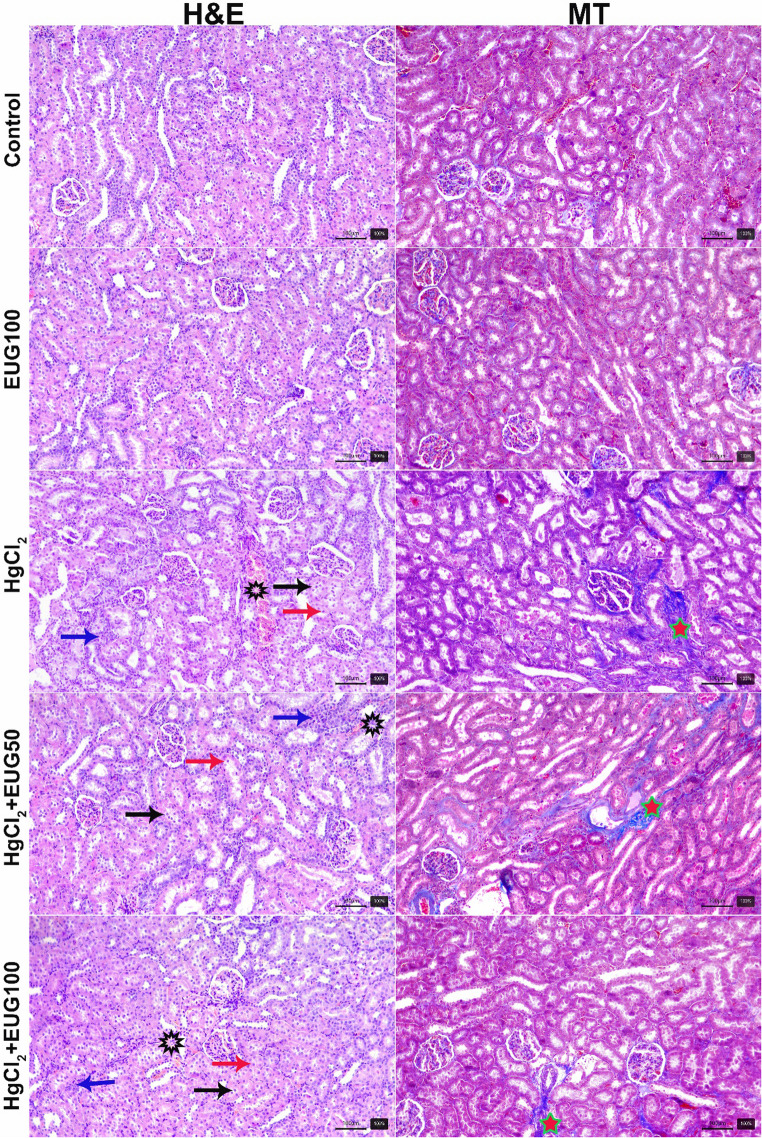
Hematoxylin-Eosin (H&E) and Masson’s Trichrome (MT) stained images of kidney tissue. In the H&E stain, black arrows indicate degenerative changes in tubular epithelial cells, red arrows indicate necrotic areas, blue arrows indicate foci of nephritis, and black stars indicate areas of hemorrhage. In MT staining, green stars indicate areas of fibrosis. Staining: H&E and MT; objective: 10×; scale bar: 100 μm

**Fig. 4:**
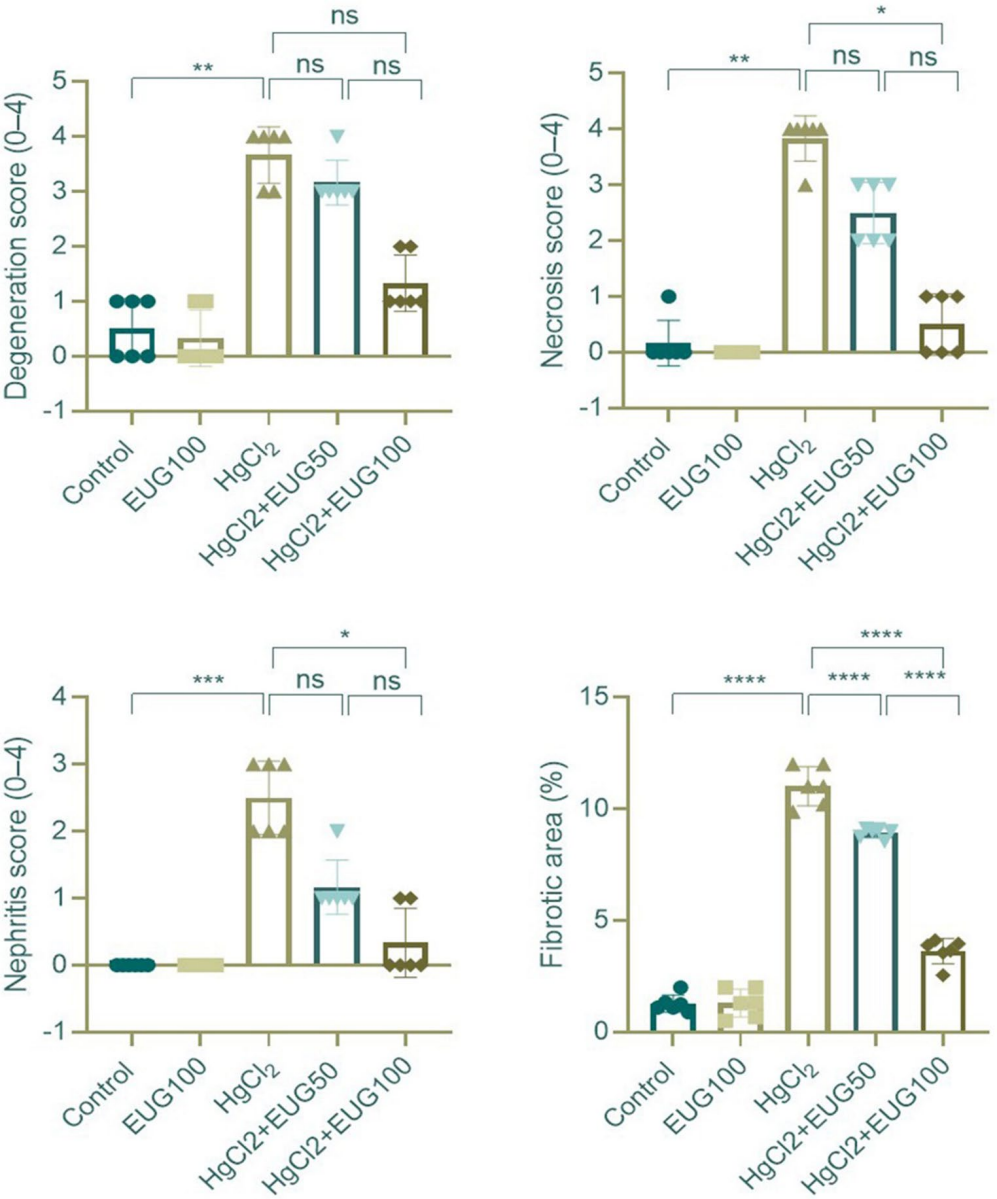
Histopathological findings including degeneration, necrosis, and nephritis scores, along with the percentage of fibrotic area determined by Masson’s Trichrome staining and the statistical analysis results for these parameters. The Kruskal–Wallis test followed by Dunn’s multiple comparison test was used for the statistical evaluation of histopathological scores. One-way ANOVA and Tukey post hoc test were applied for the statistical analysis of fibrotic area percentages. Histopathological data are presented as median (min–max), while fibrotic area data are presented as mean ± SD

In the evaluation performed with Masson’s Trichrome staining, the percentage of fibrotic area in the HgCl₂ group was found to be statistically significantly higher than in the other groups (Fig. 3). The statistical analysis results of the histopathological scores and the percentage of fibrotic area determined by Masson’s Trichrome staining are presented in Fig. 4.

### Immunohistochemical and Immunofluorescent Staining Findings

**Fig. 5:**
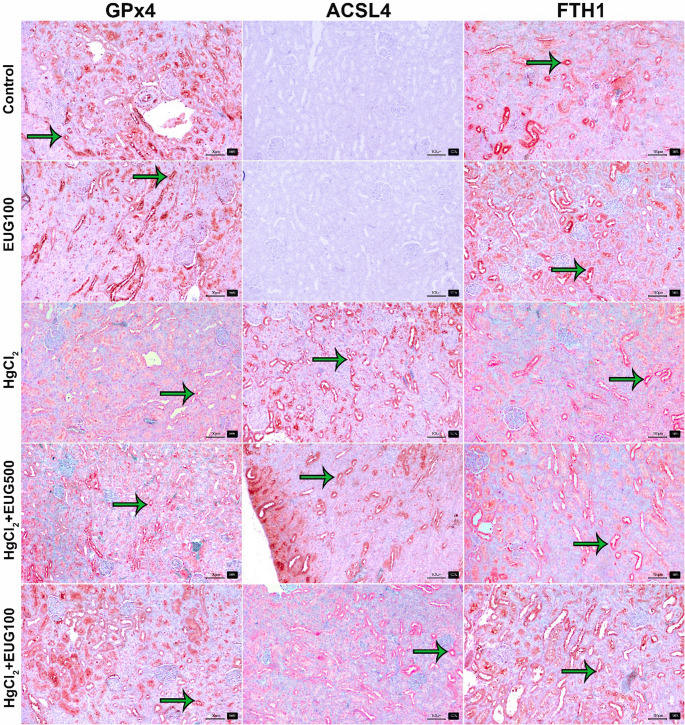
Immunoreactivity of GPx4, ACSL4, and FTH1 determined by immunohistochemical staining in kidney tissues. Green arrows indicate areas showing immunoreactive staining. Staining: IHC-P; objective: 10×; scale bar: 100 μm

**Fig. 6:**
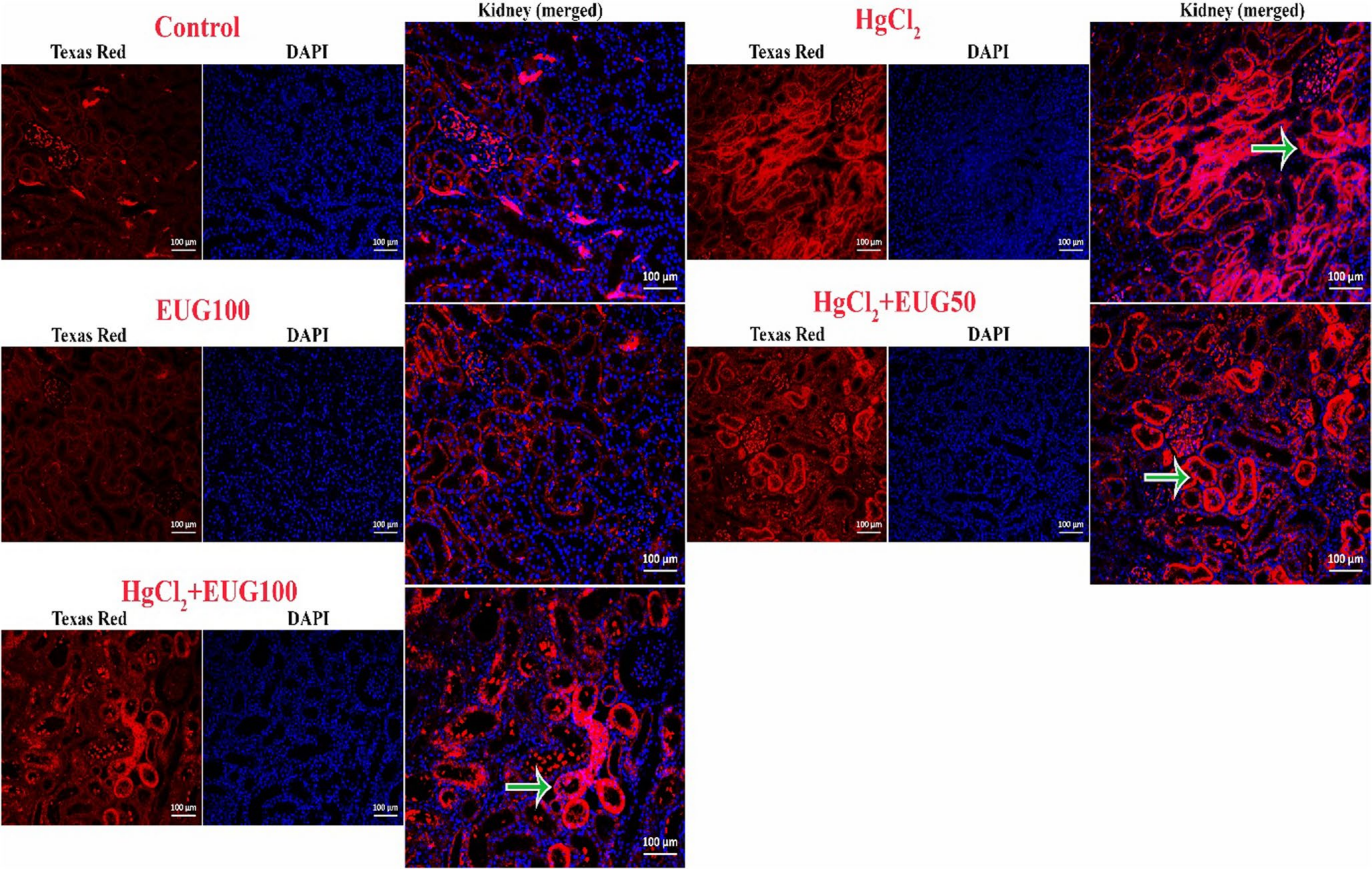
Immunoreactivity of 8-OHdG detected by immunofluorescent staining in kidney tissues. The red signal represents the immunoreactive fluorescent signal of 8-OHdG labeled with Texas Red, while the blue signal indicates nuclei stained with DAPI. The composite images were obtained by merging the Texas Red and DAPI channels. Staining: IF; objective: 20×; scale bar: 100 μm

**Fig. 7:**
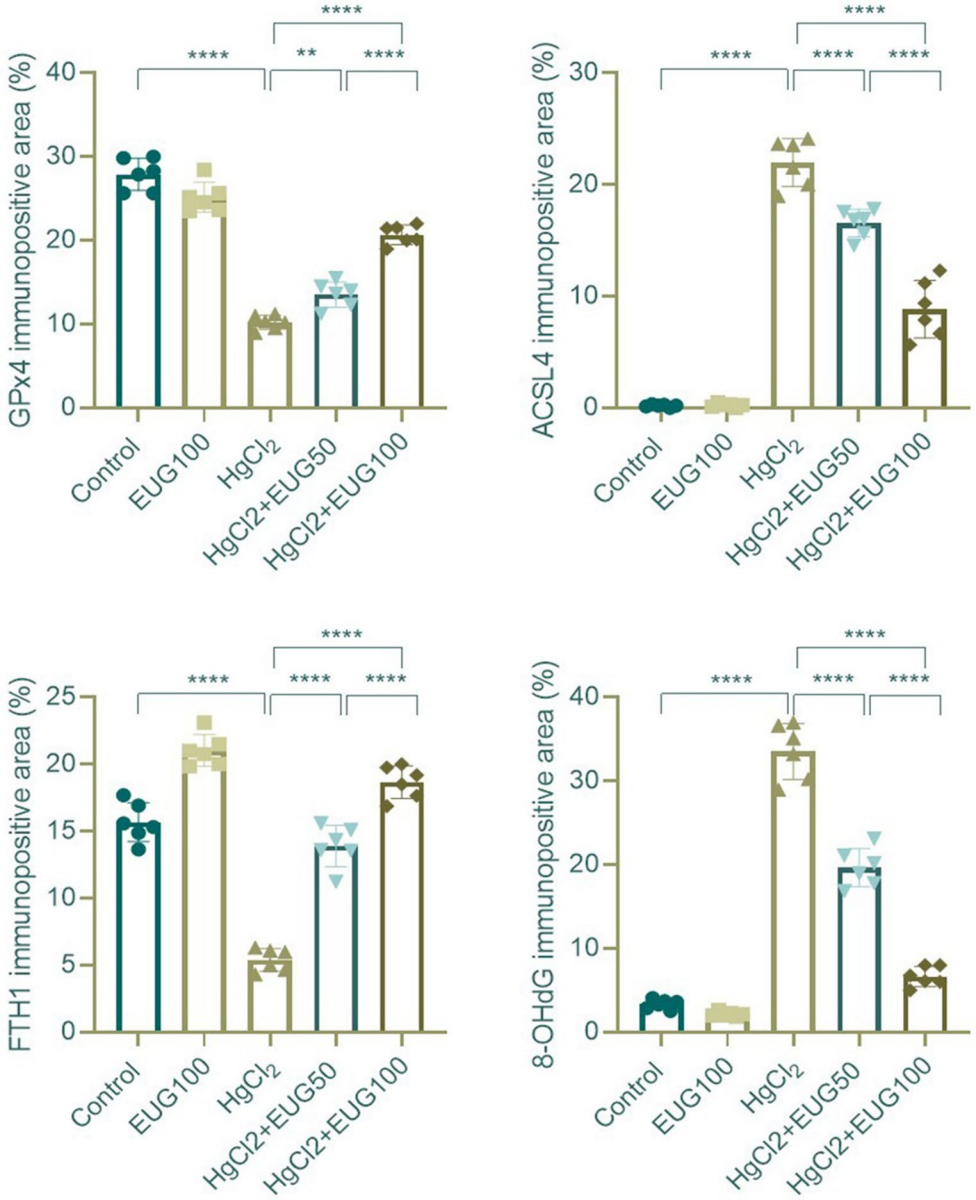
ImageJ-based semi-quantitative analysis results of the GPx4, ACSL4, FTH1, and 8-OHdG immunoreactive area percentages in IHC and IF staining. One-way ANOVA and Tukey post hoc test were used in statistical analyses. Data are presented as mean ± SD

**Fig. 8:**
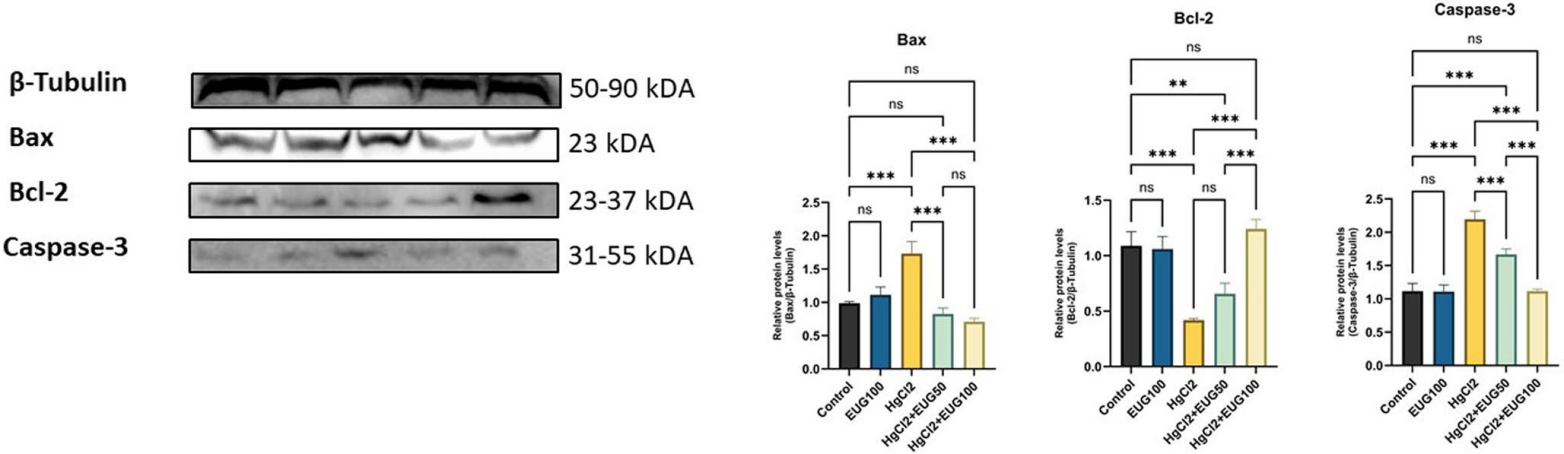
Bax, Bcl-2, and Caspase-3 protein expression levels in kidney tissue across the experimental groups. Protein levels were determined by Western blot analysis and normalized to β-tubulin. Densitometric analyses are presented as relative protein expression levels. Data are presented as mean ± SD (*n* = 6). Statistical analysis was performed using one-way ANOVA followed by Tukey’s post hoc test. ***p < 0.001, **p < 0.01; ns, not significant

In immunohistochemical (IHC) and immunofluorescent (IF) staining of kidney tissue samples, distinct immunoreactive staining was observed for GPx4 (Fig. 5) and FTH1 (Fig. 5) in the control group, while weak staining was observed for ACSL4 (Fig. 5) and 8-OHdG (Fig. 6). In the HgCl₂ group, GPx4 and FTH1 immunoreactivity decreased, whereas ACSL4 and 8-OHdG immunoreactivity increased significantly. In the groups treated with both HgCl₂ and EUG, GPx4 and FTH1 immunoreactivity increased, while ACSL4 and 8-OHdG immunoreactivity decreased. The semi-quantitative analysis results of the IHC and IF staining and the statistical evaluations of these data are presented in Fig. 7.

### Effects of HgCl₂ and EUG on Apoptosis-Related Protein Expression in Kidney Tissue

Protein expression levels associated with apoptotic pathways in kidney tissue were evaluated by Western blot analysis. In the HgCl₂-treated group, Bax and Caspase-3 protein levels were significantly increased compared to the control group, whereas anti-apoptotic protein Bcl-2 expression was significantly decreased (*p* < 0.001). In the group administered EUG alone, Bax, Bcl-2, and Caspase-3 protein levels were similar to those in the control group, and no statistically significant difference was found between the groups (ns) (Fig. 8).

In groups treated with EUG50 in combination with HgCl₂, a significant decrease in Bax and Caspase-3 protein levels was observed compared to the HgCl₂ group (*p* < 0.001), while no significant increase was observed in Bcl-2 protein levels (ns). A more pronounced trend toward improvement in apoptotic protein expression was observed in parallel with the increase in EUG dose (*p* < 0.001, Fig. 8).

### Effects of HgCl₂ and EUG on Autophagy-Related Gene Expression in Kidney Tissue

**Fig. 9:**
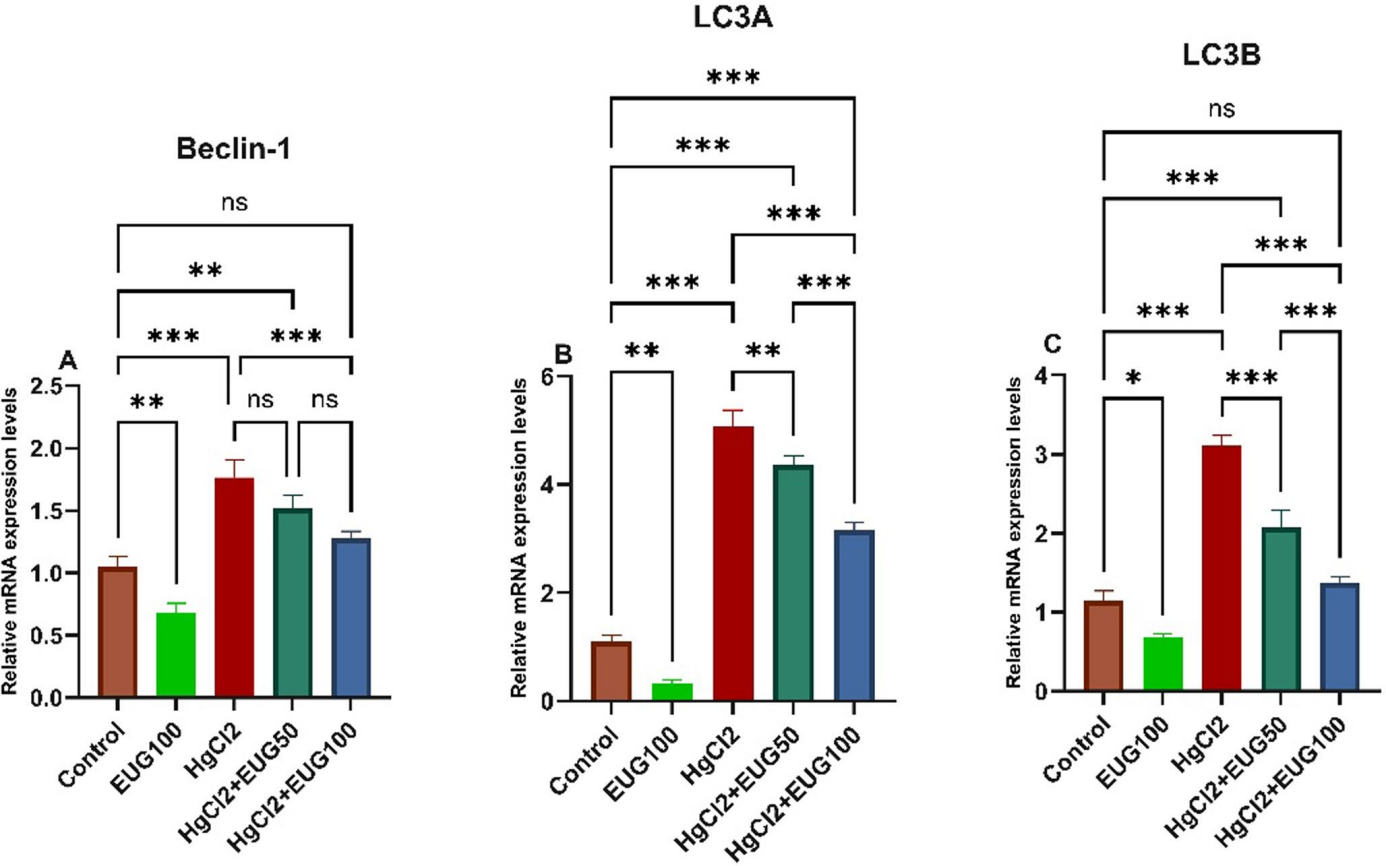
Beclin-1 (**A**), LC3A (**B**), and LC3B (**C**) mRNA expression levels in kidney tissue across the experimental groups. Gene expression levels were determined by RT-qPCR and normalized to the control group. Data are presented as mean ± SD (*n* = 6). Statistical analysis was performed using one-way ANOVA followed by Tukey’s post hoc test. ***p < 0.001, **p < 0.01, *p < 0.05; ns, not significant

The expression levels of autophagy-related genes in kidney tissue were evaluated by RT-qPCR analysis. In the HgCl₂ group, the mRNA expression levels of Beclin-1, LC3A, and LC3B were found to be significantly elevated compared to the control group (*p* < 0.001, Fig. 9).

In groups treated with HgCl₂ and EUG50, a significant decrease in LC3A (*p* < 0.01) and LC3B (*p* < 0.001) mRNA expression levels was observed compared to the HgCl₂ group. Autophagy-related gene expression levels in the HgCl₂+EUG100 group showed a marked suppression trend compared to the HgCl₂ group (*p* < 0.001, Fig. 9).

### Effects of HgCl₂ and EUG on Endoplasmic Reticulum Stress–Related Gene Expression in Kidney Tissue

**Fig. 10:**
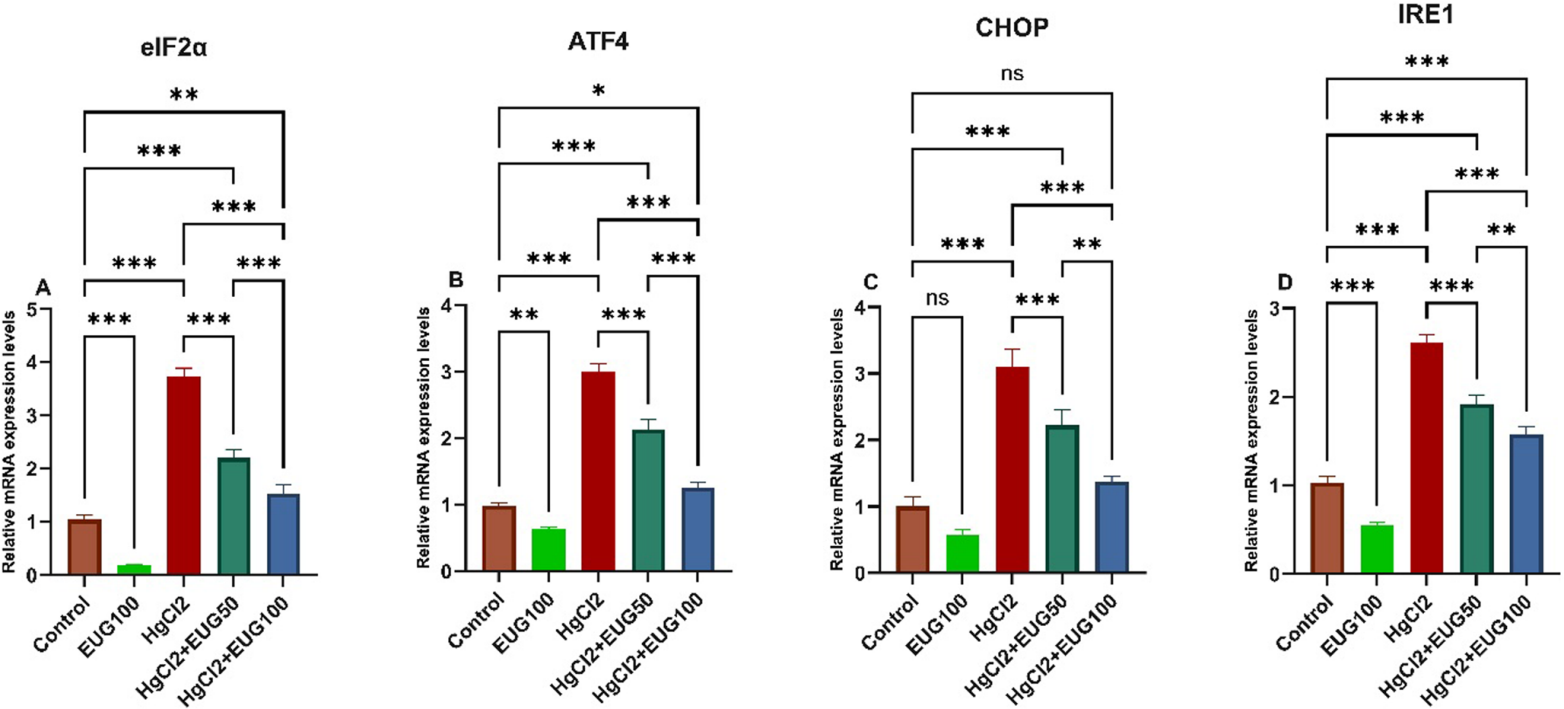
eIF2α (**A**), ATF4 (**B**), CHOP (**C**), and IRE1 (**D**) mRNA expression levels in kidney tissue across the experimental groups. Gene expression levels were determined by RT-qPCR and normalized to the control group. Data are presented as mean ± SD (*n* = 6). Statistical analysis was performed using one-way ANOVA followed by Tukey’s post hoc test. ***p < 0.001, **p < 0.01, *p < 0.05; ns, not significant

The mRNA expression levels of genes associated with endoplasmic reticulum (ER) stress in kidney tissue were evaluated by RT-qPCR analysis. In the HgCl₂-treated group, the mRNA expression levels of eIF2α, ATF4, CHOP, and IRE1 were found to be significantly elevated compared to the control group (*p* < 0.001, Fig. 10).

In groups treated with HgCl₂ and EUG50, a significant decrease in eIF2α, ATF4, CHOP, and IRE1 mRNA expression levels was observed compared to the HgCl₂ group (*p* < 0.001). When the data for the HgCl₂ + EUG100 group were compared with those for the HgCl₂ group, the statistical difference appeared to be the same, but the suppression was observed to be stronger (*p* < 0.001, Fig. 10).

## Discussion

In this study, it was demonstrated that HgCl₂ exposure does not only cause oxidative stress-induced damage in kidney tissue; rather, it leads to a multi-faceted toxicity profile in which inflammation, ferroptosis, endoplasmic reticulum (ER) stress, autophagy, and apoptosis are simultaneously activated. The biochemical, histopathological, and molecular findings indicate that these pathological processes do not occur independently but rather progress through mutual interaction. Eugenol administration, particularly at the higher dose, markedly attenuated this multi-layered damage response by restoring oxidative balance, limiting the inflammatory response, and alleviating the activation of cellular stress and cell death pathways. Oxidative stress is a key upstream driver in HgCl₂ nephrotoxicity. Thus, the modulation of ferroptosis- and ER stress–related markers by eugenol is most likely a coordinated downstream consequence of improved redox homeostasis rather than direct pathway-specific inhibition.

Following HgCl₂ exposure, the increase in MDA levels in kidney tissue, along with a significant decrease in GSH concentration and SOD protein levels, indicates that HgCl₂ activates oxidative stress as an early and central trigger by disrupting redox homeostasis in its nephrotoxicity. It has been demonstrated in various experimental models that HgCl₂ targets the mitochondrial electron transport chain, leading to electron leakage and excessive reactive oxygen species (ROS) production; in parallel, endogenous antioxidant defense systems are suppressed [31, 32]. Increased ROS production and depletion of antioxidant capacity cause MDA accumulation by increasing lipid peroxidation, creating an oxidative environment that threatens cellular integrity. Indeed, similarly, in heavy metal-induced nephrotoxicity models, oxidative stress has been reported to be a common starting point that triggers inflammation, endoplasmic reticulum stress, and cellular death pathways [33, 34]. The application of eugenol, particularly at a dose of 100 mg/kg, reduced lipid peroxidation, thereby reducing MDA levels and partially restoring redox balance in kidney tissue by increasing GSH and SOD levels. This protective effect is consistent with previous studies reporting that eugenol supports endogenous antioxidant defense mechanisms in addition to directly neutralizing free radicals [35, 36] and may provide a plausible mechanistic explanation for the attenuation of inflammatory and cellular stress responses observed in later sections of the study.

In this study, the significant increase in TNF-α, IL-1β, and IL-6 levels in kidney tissue following HgCl₂ administration indicates the activation of a potent proinflammatory response triggered by oxidative stress. This increase in cytokines is consistent with previous studies reporting the activation of NF-κB and related inflammatory signaling pathways in HgCl₂-induced kidney injury [26, 37]. The deepening of the proinflammatory environment not only increases tissue damage but also creates a secondary pathological step that enhances apoptotic and ER stress responses [38, 39]. In contrast, the suppression of anti-inflammatory cytokine IL-10 levels in the HgCl₂ group indicates a significant disruption of the inflammatory balance. Eugenol administration, particularly at high doses, significantly reduced TNF-α, IL-1β, and IL-6 levels while increasing IL-10 levels, thereby restoring the balance of the inflammatory response. These findings demonstrate that eugenol not only suppresses proinflammatory signals but also limits the severity of inflammation in HgCl₂-induced kidney damage by supporting anti-inflammatory regulatory mechanisms.

The structural counterpart of oxidative stress and inflammatory response detected at the molecular and biochemical levels in kidney tissue has been clearly demonstrated in histopathological examinations. The detection of marked degenerative and necrotic changes in tubular epithelial cells, along with interstitial nephritis and widespread fibrosis areas in the HgCl₂ group, indicates that HgCl₂ causes progressive damage in renal tissue. Previous studies have also reported that HgCl₂ exposure is characterized by tubular damage and an increased fibrotic response [40, 41]. The increased ratio of fibrotic areas determined by Masson’s Trichrome staining supports the notion that inflammation has transformed into a tissue response prone to chronicity. In contrast, eugenol administration, particularly at a dose of 100 mg/kg, alleviated tubular degeneration and necrosis and reduced the formation of fibrotic areas. This histopathological improvement demonstrates that eugenol’s effects in limiting oxidative stress and inflammation directly reflect in the preservation of tissue integrity and that there is a strong relationship between biochemical findings and structural outcomes.

One of the most striking findings of this study is the significant regulation of molecular markers associated with ferroptosis in HgCl₂-induced kidney injury. In the HgCl₂-treated group, decreased GPx4 and FTH1 immunoreactivity, along with increased ACSL4 expression and levels of the DNA oxidative damage marker 8-OHdG, strongly suggest activation of the ferroptotic cell death pathway based on lipid peroxidation. The suppression of GPx4 renders cells susceptible to ferroptosis by inhibiting the detoxification of lipid hydroperoxides, while the decrease in FTH1 levels paves the way for an increase in the free iron pool and deepening oxidative damage via Fenton reactions [42, 43]. In parallel, increased ACSL4 expression accelerates the ferroptotic process by promoting the accumulation of peroxide-prone polyunsaturated fatty acids in the cell membrane [44]. Eugenol treatment, particularly at high doses, significantly elevated GPx4 and FTH1 expression while substantially reducing ACSL4 and 8-OHdG levels; thus suggesting that eugenol attenuated ferroptosis-related alterations triggered by HgCl₂. These findings reveal that eugenol limits cell death by alleviating not only classical oxidative stress–related damage but also the iron-dependent lipid peroxidation–associated alterations in HgCl₂ nephrotoxicity, adding significant mechanistic depth to the study.

In this study, it was clearly demonstrated that HgCl₂ exposure significantly activates apoptotic cell death in kidney tissue, accompanied by an increase in Bax and caspase-3 protein levels and a decrease in the expression of the anti-apoptotic protein Bcl-2. The disruption of the Bax/Bcl-2 balance is considered a fundamental mechanism that prepares the ground for increased mitochondrial membrane permeability and activation of the caspase cascade via cytochrome-c release [45]. This apoptotic response can be interpreted as one of the combined consequences of oxidative stress, inflammation, and ferroptotic damage on cellular fate, as outlined in the preceding paragraphs. Eugenol administration, particularly at increasing doses, significantly reduced Bax and caspase-3 protein levels while partially restoring Bcl-2 expression, thereby demonstrating that HgCl₂-induced mitochondrial apoptotic pathway activation was suppressed. These findings reveal that eugenol reduces cell death in kidney tissue not only by limiting oxidative damage but also by modulating mitochondrial apoptosis-related signaling.

In this study, the significant increase in Beclin-1, LC3A, and LC3B gene expression in kidney tissue following HgCl₂ exposure indicates that the autophagic response has been activated. While autophagy can initially serve as a protective adaptation mechanism under cellular stress conditions, its excessive and uncontrolled activation can promote cellular degradation and death processes [5]. The marked autophagic activation observed in the HgCl₂ group suggests that the cell is attempting to cope with its increasing damage load as a result of the oxidative stress, ferroptotic damage, and mitochondrial dysfunction described in previous sections. However, the fact that this response increased simultaneously with apoptosis and ER stress markers indicates that autophagy may contribute to the pathological process by exceeding its adaptive limits in this model. Eugenol administration significantly reduced Beclin-1 and LC3A/B mRNA expression, suggesting that the autophagy-related transcriptional response was attenuated. These findings suggest that eugenol, rather than completely inhibiting autophagy, prevents secondary excessive autophagic activation by reducing the cellular stress load, thereby contributing to the maintenance of cellular homeostasis in kidney tissue. However, it should be noted that autophagic flux was not directly assessed in the present study. Therefore, the observed changes in autophagy-related gene expression (Beclin-1, LC3A, and LC3B) may not fully reflect dynamic autophagic activity. Since autophagy is a highly dynamic and multi-step process, further studies evaluating key markers such as LC3-II/I ratio and p62/SQSTM1 protein levels are required to more accurately interpret autophagic flux in HgCl₂-induced nephrotoxicity.

This study demonstrated that HgCl₂ exposure strongly activates the endoplasmic reticulum (ER) stress response in kidney tissue, as evidenced by a marked increase in eIF2α, ATF4, CHOP, and IRE1 gene expression. The activation of ER stress via the PERK–eIF2α–ATF4–CHOP axis indicates that when protein folding capacity is exceeded, the adaptive response shifts towards apoptotic signals [46, 47]. Considering that the increase in CHOP expression is associated with Bcl-2 suppression and Bax activation, it is understood that this pathway is directly linked to the mitochondrial apoptosis activation described in the previous paragraphs [48]. However, while activation of the IRE1 pathway initially supports protein folding homeostasis, its excessive and sustained activation is known to enhance cellular death signals [49]. Eugenol treatment significantly reduced the transcriptional upregulation of ER stress–related genes, suggesting attenuation of the ER stress–related response in this model. These findings indicate that eugenol contributes to shaping the cellular fate towards adaptation rather than death by regulating the ER stress axis, which acts as a bridge between oxidative stress, ferroptosis, autophagy, and apoptosis in HgCl₂-induced nephrotoxicity.

The main limitation of the present study is the inability to evaluate key protein markers of autophagic flux, such as LC3-II/I ratio and p62/SQSTM1 expression. Since autophagy is a dynamic process, the assessment of only mRNA levels of autophagy-related genes may not fully represent autophagic activity. Therefore, further studies incorporating protein-level analyses are needed to better clarify the role of autophagy in HgCl₂-induced nephrotoxicity.

## Conclusion

The findings obtained in this study demonstrate that eugenol exhibits a significant nephroprotective effect by simultaneously modulating interrelated cellular stress and cell death pathways such as oxidative stress, inflammation, ferroptosis, endoplasmic reticulum stress, autophagy, and apoptosis in the HgCl₂-induced nephrotoxicity model. Consistent data obtained at the biochemical, histopathological, and molecular levels indicate that eugenol is not merely a symptomatic antioxidant but a multi-target protective compound that alleviates multiple interconnected stress and cell death pathways. The simultaneous evaluation of current cellular damage mechanisms such as ferroptosis and ER stress within the same experimental model strengthens the mechanistic integrity and scientific contribution of the study. In this regard, the findings provide important evidence for the potential therapeutic role of eugenol in heavy metal-induced kidney toxicity.

## Supplementary Information

Below is the link to the electronic supplementary material.

Supplementary figure 11 Supplementary File 1 (PNG 28.2 KB)

High Resolution Image (TIF 10.7 MB)

Supplementary figure 12 Supplementary File 2 (PNG 317 KB)

High Resolution Image (TIF 11.6 MB)

Supplementary figure 13 Supplementary File 3 (PNG 315 KB)

High Resolution Image (TIF 12.6 MB)

Supplementary figure 14 Supplementary File 3 (PNG 450 KB)

High Resolution Image (TIF 12.3 MB)

## Data Availability

The datasets generated and/or analyzed during the current study are not publicly available due to institutional restrictions and the need to protect laboratory raw data integrity but are available from the corresponding author on reasonable request.
