# Supplementary figures and images for: Eugenol Mitigates Mercuric Chloride–Induced Renal Injury by Attenuating Oxidative Stress, Ferroptosis, ER Stress, Apoptosis, and Autophagy
Source: Biol Trace Elem Res. 2026 Apr 29;204(8):6326–41. doi: 10.1007/s12011-026-05115-4 (PMC13369209; doi:10.1007/s12011-026-05115-4)

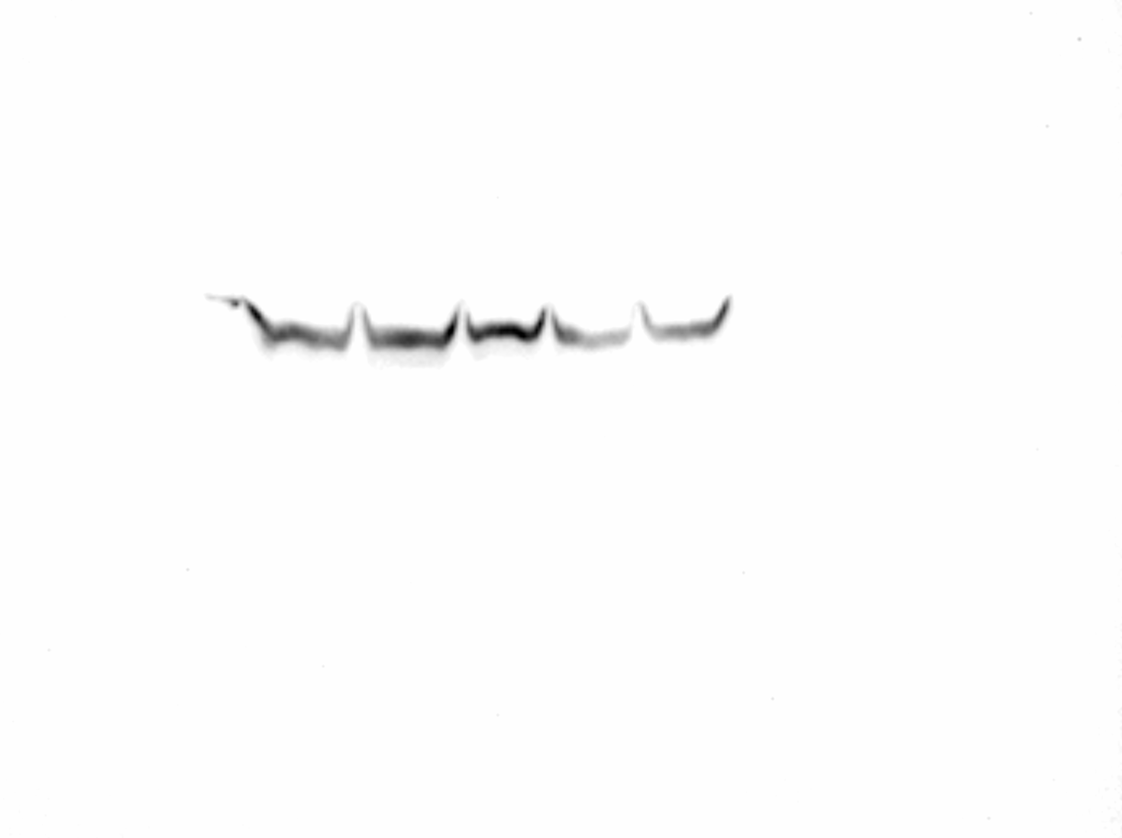

Supplement: Supplementary file 1 — (PNG 28.2 KB) [file 12011_2026_5115_Fig11_ESM.png]

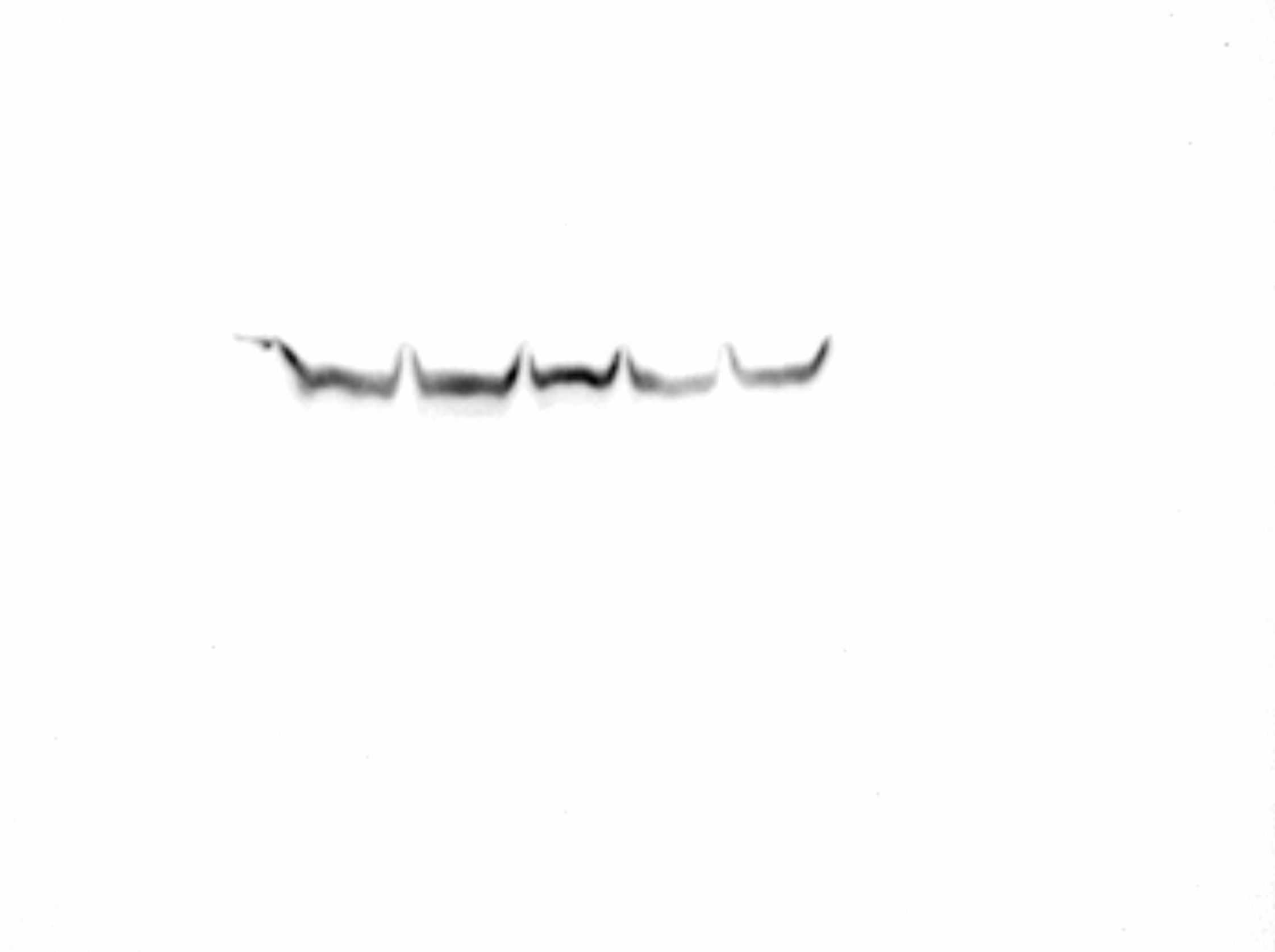

Supplement: Supplementary file 2 — High Resolution Image (TIF 10.7 MB) [file 12011_2026_5115_MOESM1_ESM.tif]

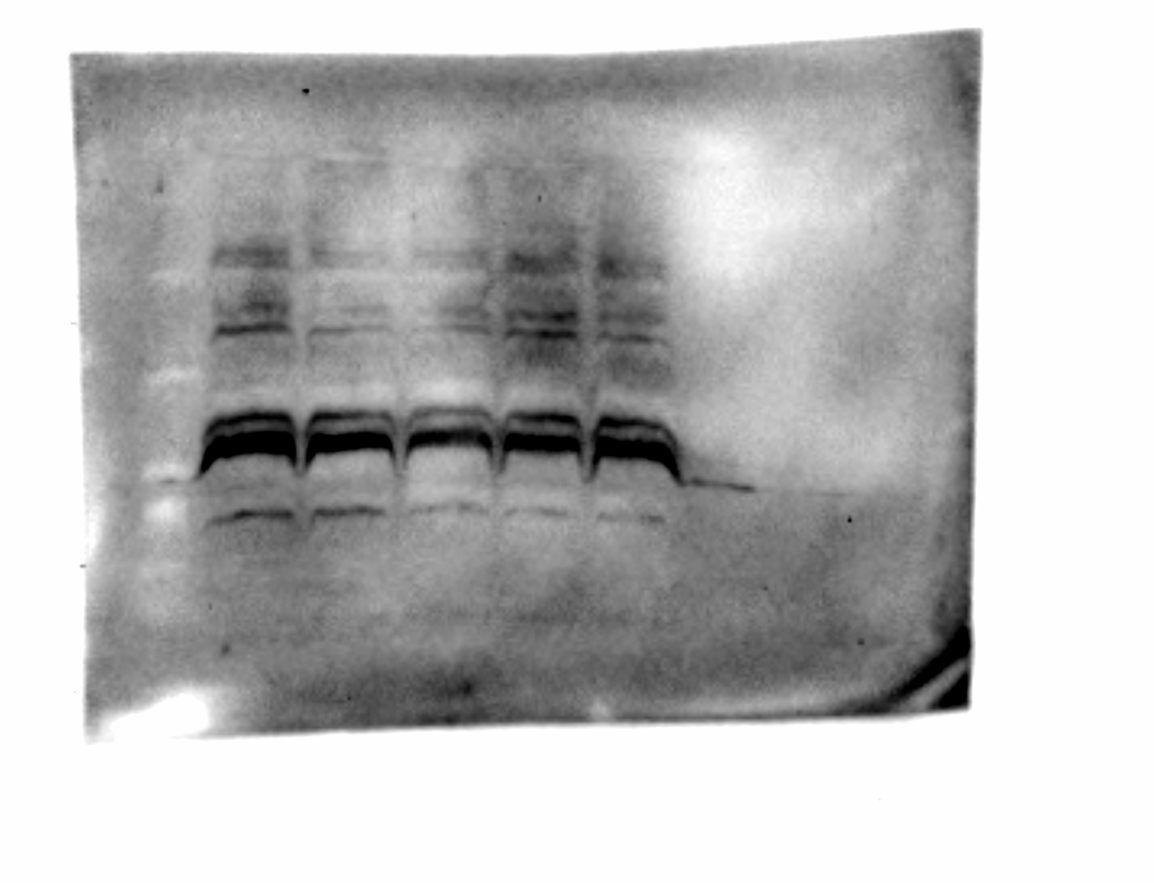

Supplement: Supplementary file 3 — Supplementary File 2 (PNG 317 KB) [file 12011_2026_5115_Fig12_ESM.png]

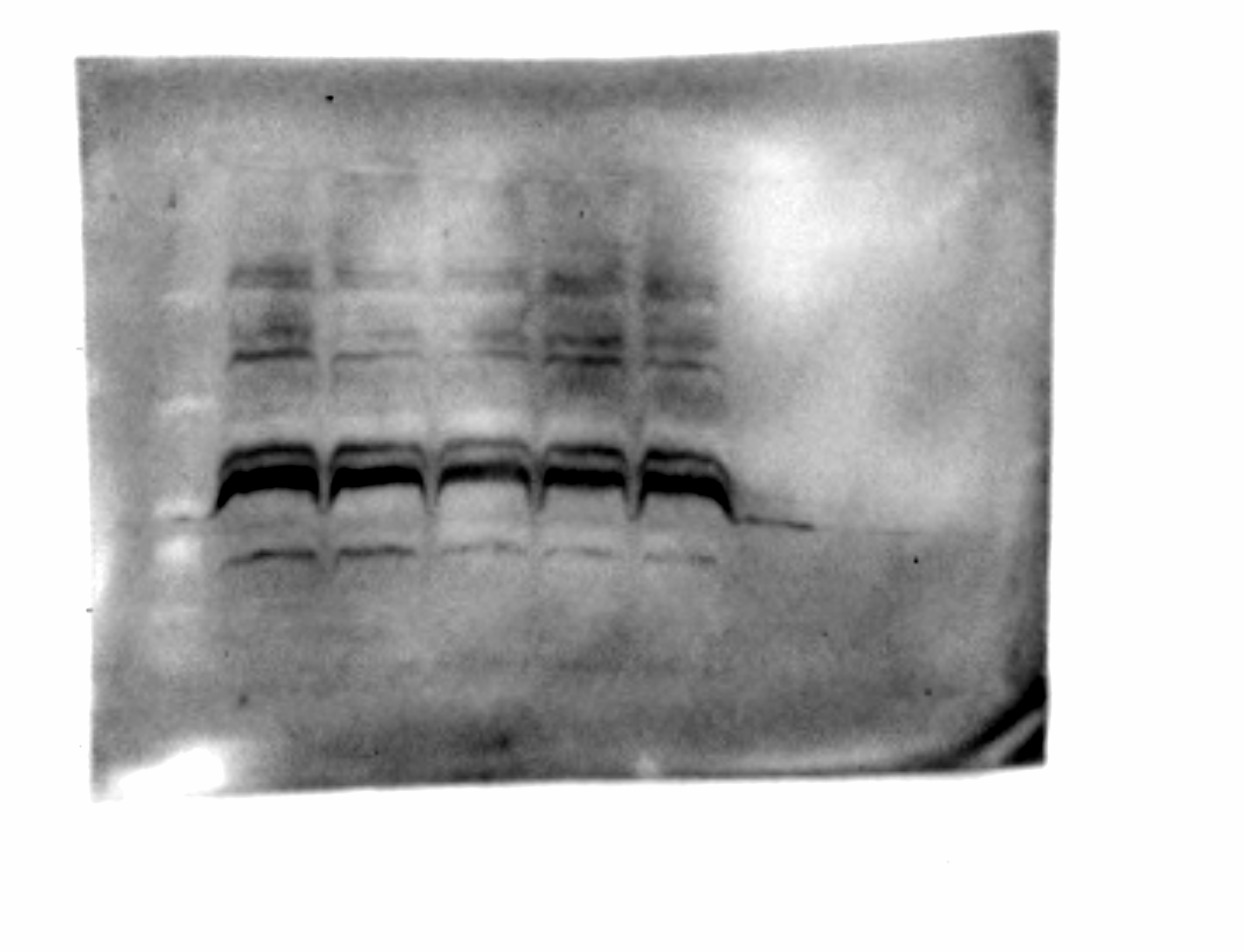

Supplement: Supplementary file 4 — High Resolution Image (TIF 11.6 MB) [file 12011_2026_5115_MOESM2_ESM.tif]

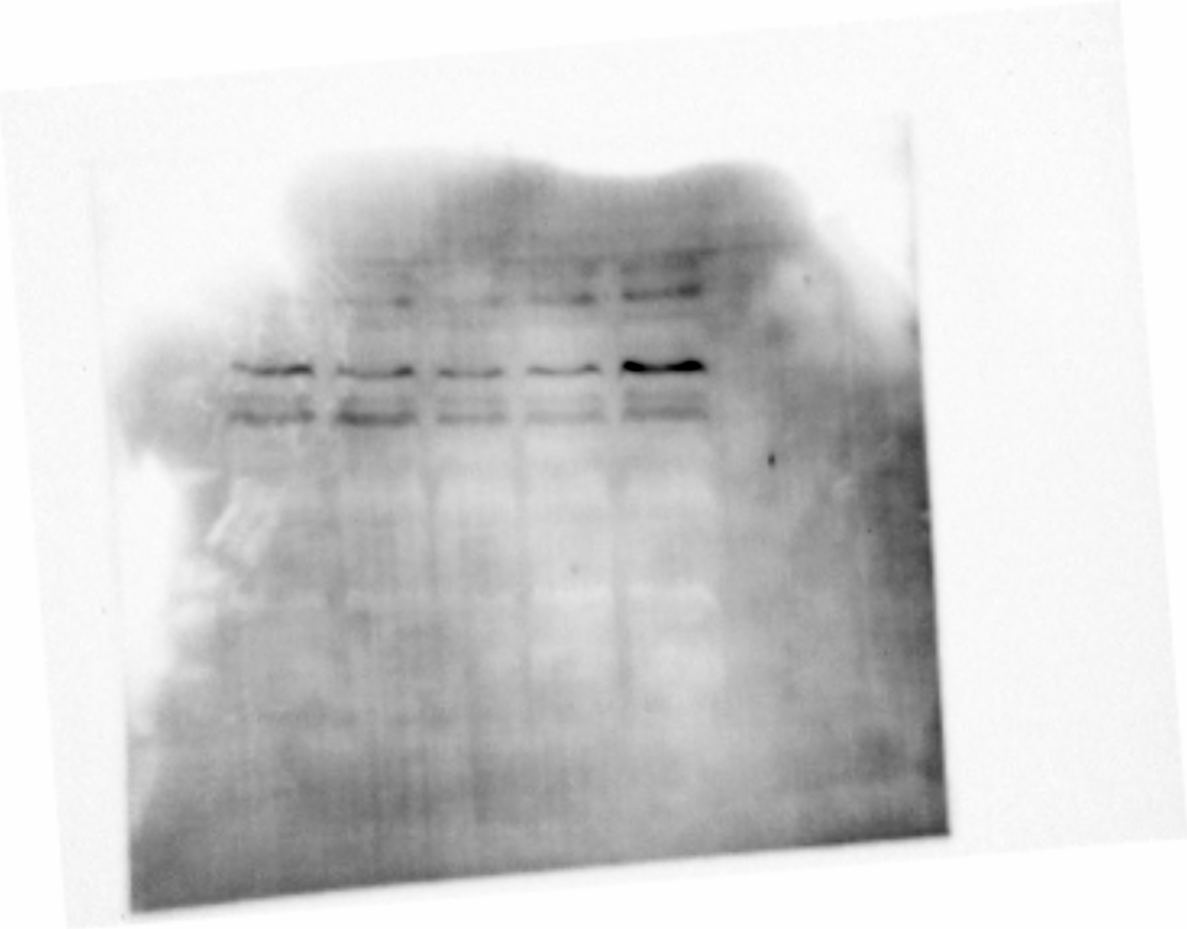

Supplement: Supplementary file 5 — Supplementary File 3 (PNG 315 KB) [file 12011_2026_5115_Fig13_ESM.png]

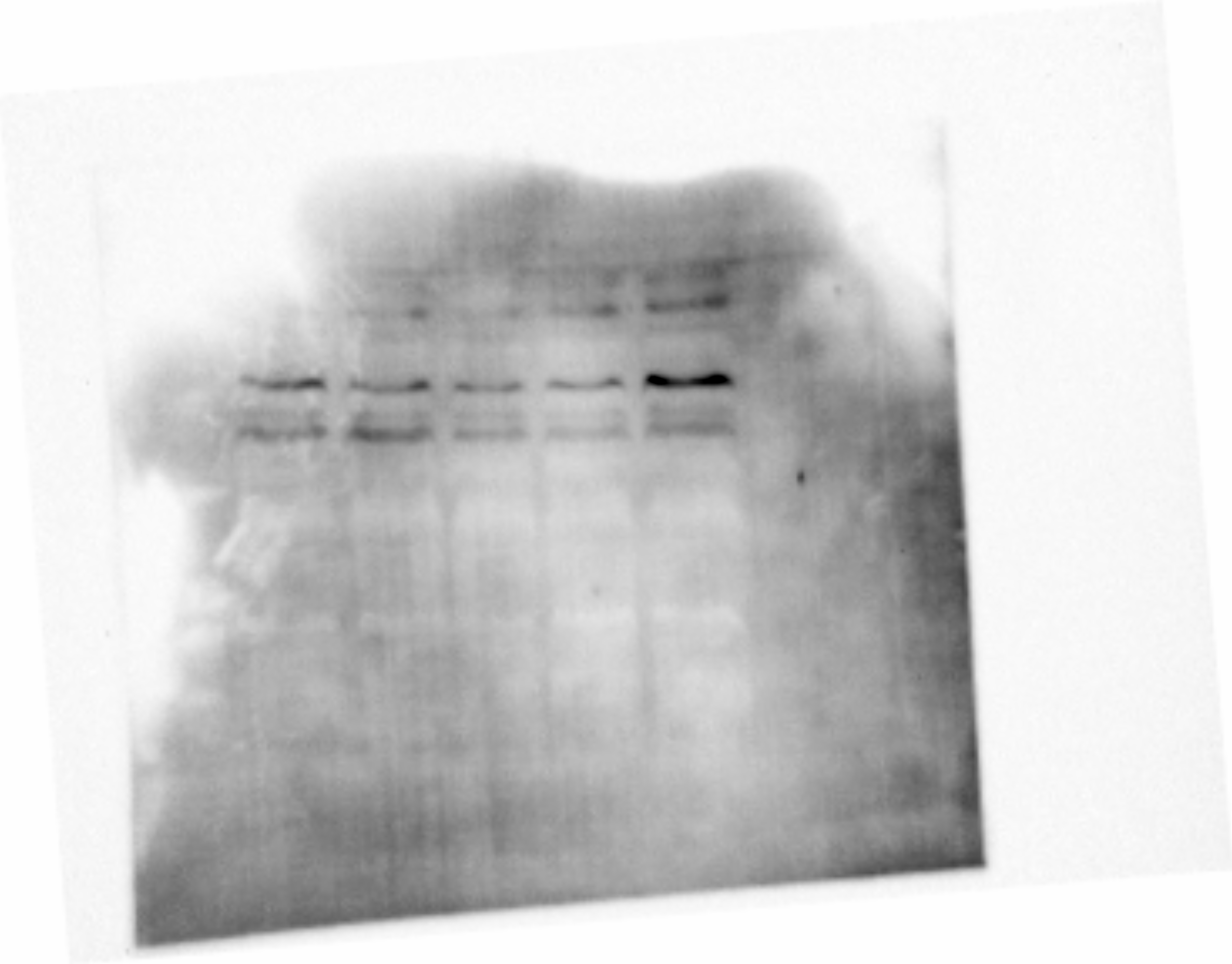

Supplement: Supplementary file 6 — High Resolution Image (TIF 12.6 MB) [file 12011_2026_5115_MOESM3_ESM.tif]

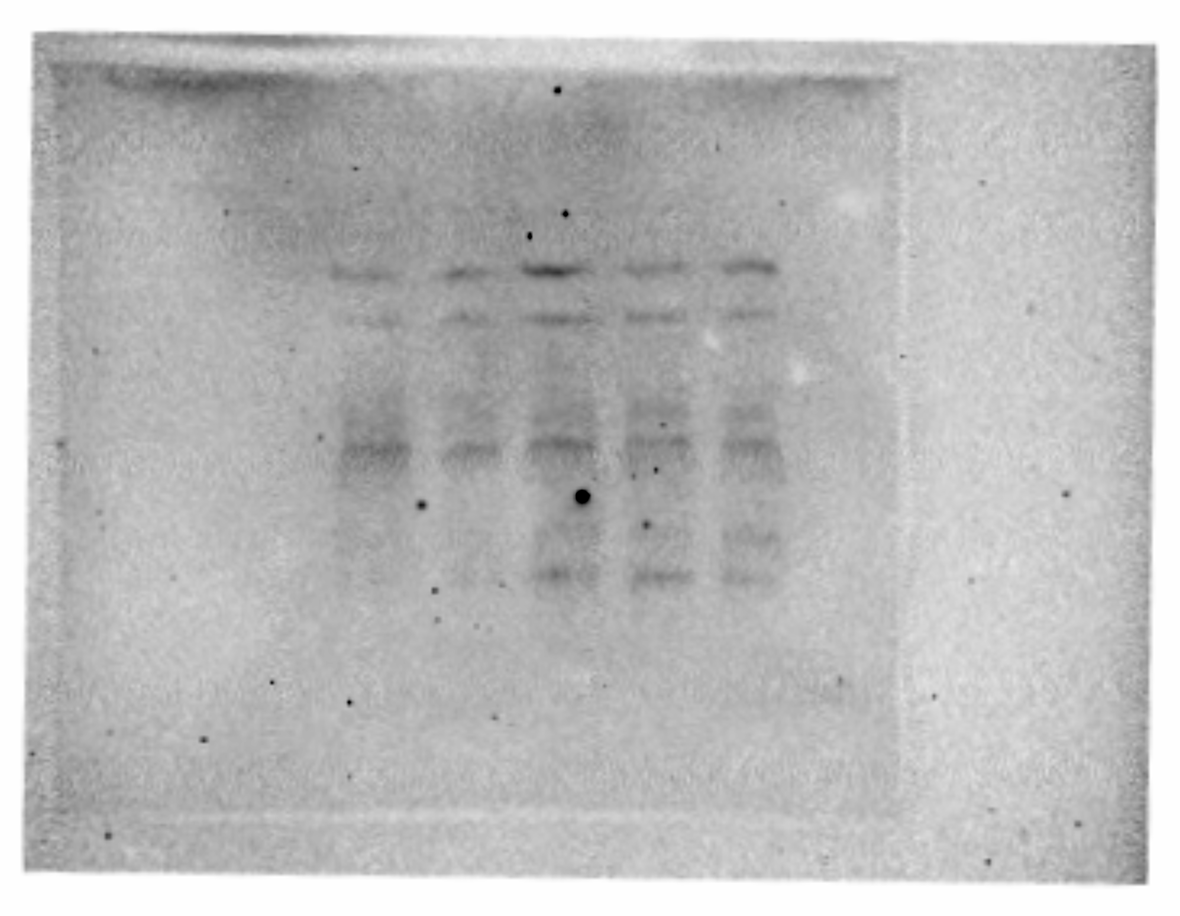

Supplement: Supplementary file 7 — Supplementary File 3 (PNG 450 KB) [file 12011_2026_5115_Fig14_ESM.png]

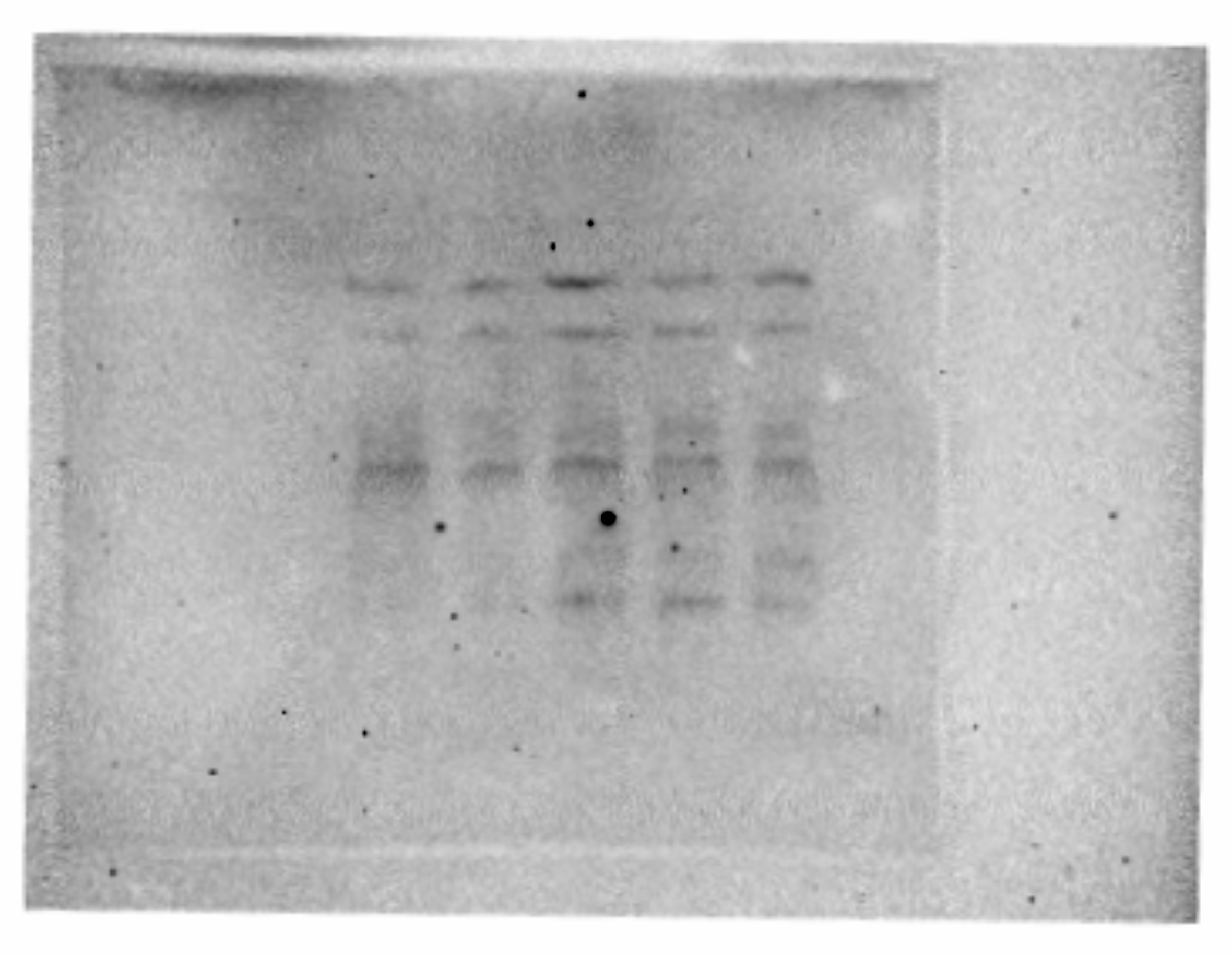

Supplement: Supplementary file 8 — High Resolution Image (TIF 12.3 MB) [file 12011_2026_5115_MOESM4_ESM.tif]
